# Fuzzy relationships among plant dispersal mechanisms, syndromes, and animal vectors

**DOI:** 10.1002/ecy.70450

**Published:** 2026-07-08

**Authors:** Casper H. A. van Leeuwen, Ruben Heleno, Andy J. Green, Esther Sebastián‐González, José Miguel Costa, Christophe Baltzinger, Evan Fricke, Ádám Lovas‐Kiss, Irene Castañeda, Isabel Donoso, Alistair G. Auffret, Sara B. Mendes

**Affiliations:** ^1^ Department of Ecology, Radboud Institute for Biological and Environmental Sciences, Faculty of Science Radboud University Nijmegen The Netherlands; ^2^ Centre for Functional Ecology, Associate Laboratory TERRA, Department of Life Sciences University of Coimbra, Calçada Martim de Freitas Coimbra Portugal; ^3^ Department of Conservation Biology and Global Change Estación Biológica de Doñana (EBD), CSIC Seville Spain; ^4^ Department of Ecology University of Alicante Alicante Spain; ^5^ ‘Ramón Margalef’ Multidisciplinary Institute for the study of the Environment University of Alicante Alicante Spain; ^6^ Research Unit Forest Ecosystems INRAE Val de Loire Nogent‐sur‐Vernisson France; ^7^ Department of Civil and Environmental Engineering Massachusetts Institute of Technology Cambridge Massachusetts USA; ^8^ MTA‐HUN‐REN Centre for Ecological Research, Momentum Dispersal Ecology Research Group Debrecen Hungary; ^9^ Department of Planetary Health, Faculty of Health Sciences, Institute of One Health University of Debrecen Debrecen Hungary; ^10^ Ecology and Genetics of Conservation and Restoration, UMR INRA 1202 BIOGECO, Université de Bordeaux Pessac France; ^11^ BC3 Basque Centre for Climate Change, Scientific Campus of the University of the Basque Country Sede Building 1 Leioa Spain; ^12^ Ikerbasque, Basque Foundation for Science Bilbao Spain; ^13^ Department of Ecology Swedish University of Agricultural Science Uppsala Sweden; ^14^ Department of Biodiversity, Macroecology and Biogeography, Faculty of Forest Sciences and Forest Ecology University of Göttingen Göttingen Germany

**Keywords:** biotic interactions, dry fruit, ecological networks, endozoochory, epizoochory, fleshy‐fruit, myrmecochory, seed dispersal syndromes, synzoochory, traits, zoochory

## Abstract

Knowledge of which animal clades and dispersal mechanisms contribute to seed dispersal is critical for understanding the ecology and evolution of plant communities. However, a broad understanding is limited by a focus on single clades and mechanisms. Here, we analyzed a Europe‐wide multi‐clade seed dispersal dataset with interactions among 447 animal species and 1901 plant species via four dispersal mechanisms: endozoochory (ingestion followed by egestion), epizoochory (external attachment), synzoochory (actively carried by vertebrates), and myrmecochory (dispersal by ants). We aimed to (1) characterize the relative importance of animal dispersal clades and biotic dispersal mechanisms; (2) evaluate links among clades and mechanisms; (3) compare dispersal of dry‐ and fleshy‐fruited diaspores; and (4) evaluate the predictive power of seed dispersal syndromes with empirical data on observed dispersal mechanisms. This revealed that endozoochory by birds and mammals encompasses 90% of plant–animal interaction records. Mammal species tend to have more plants depending upon their dispersal services; however, due to the larger diversity of bird species, more plants depend upon the cumulative services of birds (followed by mammals, insects, and reptiles). Plants with fleshy fruits comprise only 15% of all plant species dispersed by animals but had more animal partners per plant species than plants with dry fruits. Fleshy‐fruited plant species were more specifically selected by dispersers, while selection of dry‐fruited plants depended more on their relative abundance. Different animal clades tend to disperse plant species via different mechanisms. Endozoochory is the most recorded interaction for birds, mammals, and reptiles. Epizoochory is almost exclusively carried out by mammals, with a negligible role of birds, insects, and reptiles. Synzoochory is performed chiefly by ants (via myrmecochory), followed by birds and then mammals. Predicting vector importance by assigning dispersal syndromes based on diaspore traits (including endozoochorous, epizoochorous, synzoochorous, and myrmecochorous syndromes) only moderately predicted empirically observed dispersal mechanisms. Animals disperse many more species than inferred from diaspore traits, including plants with abiotic syndromes and plants without clearly visible morphological traits. Our results suggest that different animal clades perform complementary dispersal services via multiple mechanisms to plant species with fleshy as well as dry fruits.

## INTRODUCTION

Seed dispersal is a critical process for vegetation dynamics and consequently for ecosystem functioning (Dirzo et al., 2014; Ozinga et al., 2009). Approximately half of all plants have been estimated to be dispersed by animals (i.e., zoochory), a proportion that could be even higher (Rogers et al., 2021). This implies that ongoing defaunation can have cascading negative effects on the capacity of plant species to disperse (Fricke et al., 2022). Animals can provide dispersal, often over long distances (Bullock et al., 2017), which is essential for plants to modify their distributions and maintain their populations in response to climate change and increasing fragmentation of the landscape (Lovas‐Kiss et al., 2023; Nowak et al., 2022; Viana, 2017). Understanding and predicting the mechanisms by which animal vectors disperse seeds can therefore help us bend the curve of biodiversity loss (Leclère et al., 2020).

Multiple animal clades, including insects, birds, mammals, and reptiles, contribute to plant seed dispersal across the globe (Fricke et al., 2025; Mendes et al., 2024). Each animal species interacts with specific plant species and may do so via four key dispersal mechanisms: endozoochory (ingestion and subsequent egestion), epizoochory (transport via attachment) and synzoochory (i.e., when seeds or fruits are carried intentionally by animals, for example for hoarding), of which the latter is separately referred to as myrmecochory if carrying is conducted by ants (van der Pijl, 1969). Historically, however, most empirical seed dispersal studies focus on a single animal clade (e.g., only birds, Sebastián‐González et al., 2020), on specific plant groups (e.g., only fleshy fruits, Quintero et al., 2024) and/or on specific dispersal mechanisms (e.g., only endozoochory, van Leeuwen et al., 2023). While these focused studies provide specific in‐depth knowledge, they do not allow an understanding of the relative importance of different animal clades and dispersal mechanisms securing the seed dispersal services for entire plant communities. An important way forward is to better integrate field data from multiple animal clades and plant species with contrasting seed morphologies. This can help to better understand the relationships among animal species, plant species and the specific dispersal mechanisms that link them (Donatti et al., 2011; Escribano‐Avila et al., 2018).

It has been a long‐standing endeavor in the ecological literature to classify these plant‐disperser relationships. A primary example of this is the definition of ‘seed dispersal syndromes’, that is, groups of morphological diaspore traits assumed to facilitate dispersal through a specific mechanism (Vargas et al., 2023). These classifications, first formulated by van der Pijl (1969), are based on assumptions that diaspore traits such as fleshy pulp provide an adaptive advantage for endozoochory because they reward animal vectors, diaspores with hooks facilitate attachment to mammal fur, low‐density tissues increase buoyancy for water dispersal, and plumes decrease terminal velocity for wind dispersal. Despite the usefulness of diaspore traits to understand why certain dispersal mechanisms might be favored over others (Arjona et al., 2018; Vargas et al., 2014), these syndromes cannot explain, and should not be assumed to explain, the dispersal of every single diaspore (Thomson et al., 2010; Vargas et al., 2023). Indeed, there are many well‐known examples of seeds that are dispersed by mechanisms that were not predicted based on diaspore traits (González‐Varo et al., 2024; Higgins et al., 2003; Vargas et al., 2012). Such events include large amounts of dry seeds identified in the feces of herbivores or waterbirds (Albert, Mårell, et al., 2015; Green et al., 2022; Rojas et al., 2022; Wasowicz et al., 2025), the external adhesion of diaspores without hooks or other fixating appendages (Brochet et al., 2010; Costa et al., 2014), and even the colonization of remote islands by diaspores without any visible adaptation to long distance seed dispersal (Heleno & Vargas, 2015). However, without rigorous analysis contrasting predicted and observed dispersal mechanisms across multiple clades, it is impossible to quantify to what extent seed dispersal syndromes actually predict the role of animal seed dispersal in nature.

Here, we used a Europe‐wide seed dispersal database reporting empirical dispersal events between 1901 plant species and 447 animal species, to address four objectives. First, we characterized the relative importance of animal dispersal clades (birds, mammals, reptiles, and insects) and the relative importance of biotic dispersal mechanisms for seed dispersal in Europe. Second, we evaluated how these different animal clades contribute to different seed dispersal mechanisms (endozoochory, epizoochory, synzoochory, and myrmecochory). Third, we compared the dispersal of dry‐ and fleshy‐fruited diaspores, testing the hypothesis that a greater reward provided by fleshy fruits to animals leads to relatively higher dependency on animal vectors for their dispersal. Finally, we evaluated to what extent seed dispersal syndromes allow us to predict observed biotic dispersal mechanisms at the European scale.

## MATERIALS AND METHODS

### Literature database

Our work is focused on the extensive database compiled by Mendes et al. (2024), which includes virtually all published seed dispersal records by vertebrates (birds, mammals, reptiles, and fishes) and invertebrates (ants, beetles, gastropods) on the European continent. The geographical scope spans latitudes from 35° to 70° north, and longitudes from 10° west to 62° east (see Mendes et al., 2024 for details), including all European countries, the European part of Russia (west of the Ural Mountains), and the continental European islands, but excluding Turkey.

From this database, we retained seed dispersal records involving the best studied animal clades (birds, mammals, insects, and reptiles), which represent 99.9% of the records (31,465 out of 31,486). For other clades, such as fish and gastropods, we found only very few records. By excluding these rare observations we could analyze the data with four clear taxonomic groups, while only reducing the number of animal vector species from 455 to 447. We only selected interactions of confirmed seed dispersal (i.e., when the viability of the dispersed seeds was experimentally confirmed) and likely seed dispersal (i.e., intact seeds found in samples). Thus, interactions coded as seed predation or those where the fate of the seeds after frugivory was unknown were excluded (28.7% of the records). Finally, interactions between plants and animals were categorized into four dispersal mechanisms: (1) “Endozoochory”, if intact seeds or fruits were identified in the feces, digestive tract, regurgitated or if seeds were spat out; (2) “Epizoochory”, if seeds or fruits were found on the surface of animals (e.g., bill, fur, feathers, legs or hooves); (3) “Synzoochory”, if seeds or fruits were intentionally carried by animals (with or without seed caching), or if seeds or fruits were found in nests; and (4) “Myrmecochory”, if seeds were externally dispersed by ants. We specifically chose to keep myrmecochory separate from synzoochory in our analyses because, although myrmecochory can only be performed by ants, ants can also carry seeds lacking a myrmecochorous syndrome (see below), and seeds with such a syndrome can be transported by other animal clades besides ants. All records were resolved to the lowest possible taxonomic level, but only those records in which both interaction partners could be identified to the species level were retained for analysis. Overall, 22% of the interactions reported in the literature included supra‐specific taxa and were therefore excluded (we kept 31,465 from 40,152 records). This was furthermore important because the taxonomic resolution of animals was much more complete than that of plants, with only 1.98% (i.e., 795 records) of the interaction records excluded due to the lack of animal species‐level identification. Both native and non‐native plant and animal species were included.

### Dispersal syndromes

Information on plant traits related to specific dispersal vectors was extracted from the EuDis database Version 6.0 (Vargas et al., 2023), where each plant was coded for the presence or absence of traits related to four biotic seed dispersal syndromes: (1) endozoochorous, diaspores with nutritive tissues that foster the deliberate consumption by animals, such as a fleshy pericarp, aril or large sarcotesta (note, this syndrome was not subdivided into further syndromes sometimes proposed for different vertebrate clades, e.g. Rojas et al., 2022), (2) epizoochorous, diaspores with characteristics that promote their adhesion to an animal's body, such as hooks, spines, barbs, awns, mucilage, or resins, (3) myrmecochorous, diaspores with elaiosomes (caruncles, strophioles, and fleshy funicles), and (4) vertebrate hoarding, diaspores that present large dry fruits or seeds with abundant nutritive reserves frequently stored by vertebrates for later consumption. Note that the syndromes are solely defined by the traits of the diaspores, and not related to any potential animal dispersers. Four abiotic seed dispersal syndromes were also identified: (5) anemochorous, diaspores with hairs, tufts of cotton, plumes, pappus, a flattened rim or wings, resulting in low terminal velocity and high air resistance, (6) thalassochorous, diaspores with waxy substances or low‐density tissues that foster floatability, and the ability to survive after prolonged exposure to saltwater, (7) freshwater hydrochorous, diaspores with waxy substances or low‐density tissues that enable floatability for plants that grow in lakes and riparian habitats, and (8) ballochorous, dehiscent fruits with explosive mechanisms that eject seeds away from the mother plant (Vargas et al., 2023). Vargas et al. (2023) coded the presence of each set of traits independently, allowing for multiple syndromes in a minority of plant species (see below). For analysis, plant species considered to have none of these traits were classified as “unspecialized” in the context of dispersal syndromes. Plant species with the presence of endozoochorous traits were considered fleshy, and those without these traits were considered dry‐fruited.

### Data analyses

Qualitative plant–animal interactions were compiled into a binary incidence matrix indicating the presence/absence of an interaction between each plant species *i* and each animal species *j*. If the same interaction was described in multiple literature sources, this interaction was included only once. We calculated the following species‐level metrics: (1) Plant degree: number of animal species dispersing plant species *i* (Dormann et al., 2023); (2) Animal degree: number of plant species dispersed by animal species *j*; (3) Plant specialization (d'*_i_*): how selectively a plant species interacts with particular animal species relative to the overall availability of dispersers; ranging from 0 (minimum specialization) to 1 (maximum specialization) (Blüthgen et al., 2006); (4) Animal specialization (d'*_j_*): how selective an animal species is in interacting with particular plant species relative to the overall availability of plant species; (5) Plant species strength: a quantification of the importance of a given plant to the entire animal assemblage, calculated as the sum of the dependencies of animals on that plant. In this context “dependency” is the fraction of an individual partner's interactions with the focal species. (6) Animal species strength: a quantification of the importance of a given animal to the entire plant assemblage, calculated as the sum of the dependencies of all plants on that animal. We also calculated the cumulative species strength for each clade (i.e., birds, mammals, insects, and reptiles) by adding up all the values of species strength of the species in each clade. This gives an indication of plant dependencies on these animals and is defined as the sum of the partners “dependencies” (Bascompte et al., 2006). The network was visualized and species metrics were calculated using the R‐package *bipartite* (Dormann et al., 2023).

Differences among animal clades and between fruit types in network metrics were assessed with generalized least squares (GLS) models in R‐package *nlme* (Pinheiro et al., 2021). Three models assessed the effects of clade as a fixed categorical predictor, and three models assessed the effects of fruit type (fleshy or dry) as a fixed categorical predictor. Because sample coverage varied by factor levels (Appendix S1: Table S1), we accounted for heterogeneity of residual variances by explicitly modeling variance identity structures (varIdent) by groups. Model assumptions were evaluated by inspecting residual plots and quantile–quantile plots, and by testing residual normality using Shapiro–Wilk tests. The need to account for heterogeneous variances was confirmed by observing lower Akaike information criteria (AIC) values for GLS models with implemented variance identity structures than for models assuming homogeneous variances. Pairwise differences among clades were assessed using post hoc comparison tests with Bonferroni adjustments for multiple comparisons from R‐package *emmeans* (Lenth et al., 2019).

To evaluate how observed dispersal mechanisms related to the assigned dispersal syndromes for a given plant species, we built a two‐way contingency table summarizing the number of interactions recorded by each of the eight syndromes or ‘unspecialized’ diaspores (as rows) and each mechanism (as columns). We used omnibus chi‐square tests of independence to compare expected versus observed syndromes. All analyses were performed in R version 4.2.2 (R Core Team, 2025).

## RESULTS

### The contributions of dispersal clades and mechanisms

The database included interactions among 1901 plant species and 447 animal species. Of these plants, 64% (1208 species) were recorded as dispersed by 283 bird species, and 61% (1156) by 69 mammal species. These included 505 plant species exclusively dispersed by birds, and 432 exclusively by mammals. Ninety percent of all plant species (1718 of 1901) were dispersed by either bird and/or mammal species. Insects dispersed 24% of all plant species (455 plant species, 165 exclusively dispersed by this group, involving 84 insect species) and reptiles 4% (73 plant species, 11 exclusively, by 11 reptile species). Most plant species are dispersed via endozoochory (1646, 87%), while far fewer were dispersed via myrmecochory (451, 24%), epizoochory (426, 22%) and synzoochory (262, 14%). Note that the total percentage of plants exceeds 100% because one plant species can be dispersed by multiple mechanisms.

### Variation among clades in their interactions

**FIGURE 1:**
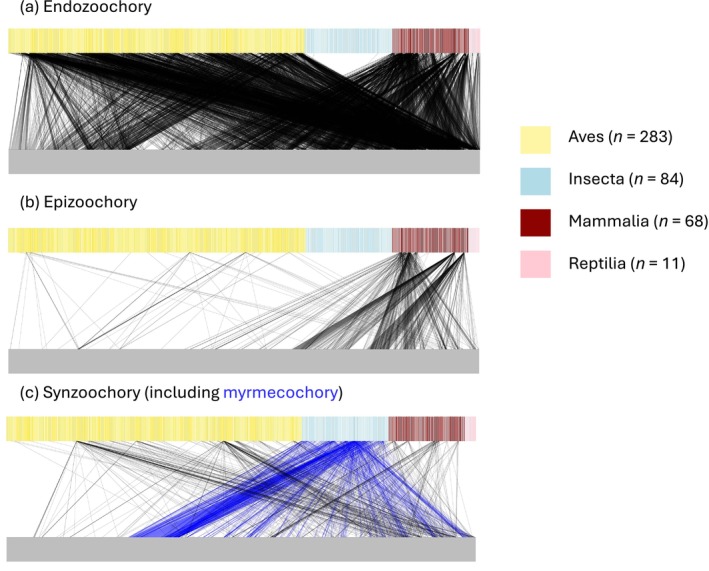
Species interactions networks via (a) endozoochory, (b) epizoochory, and (c) synzoochory (including myrmecochory, with dark blue lines) among European plant species and animals of four different clades. Each vertical bar at the top represents one animal vector species, colored by animal clades according to the legend. The lower row consists of multiple vertical bars that each represent one plant species. All plant and animal species are present in all networks, but only the interactions via one mechanism are highlighted per network.

Different animal clades were associated with different dispersal mechanisms (Figure 1). Most species dispersed by birds were dispersed via endozoochory (96%), 17% via synzoochory and 3% via epizoochory. Most species dispersed by mammals were dispersed via endozoochory (91%), 34% via epizoochory, and 8% via synzoochory. Insects dispersed species mainly via myrmecochory (99%), that is, a special form of synzoochory. All plant species dispersed by reptiles were dispersed via endozoochory.

**FIGURE 2:**
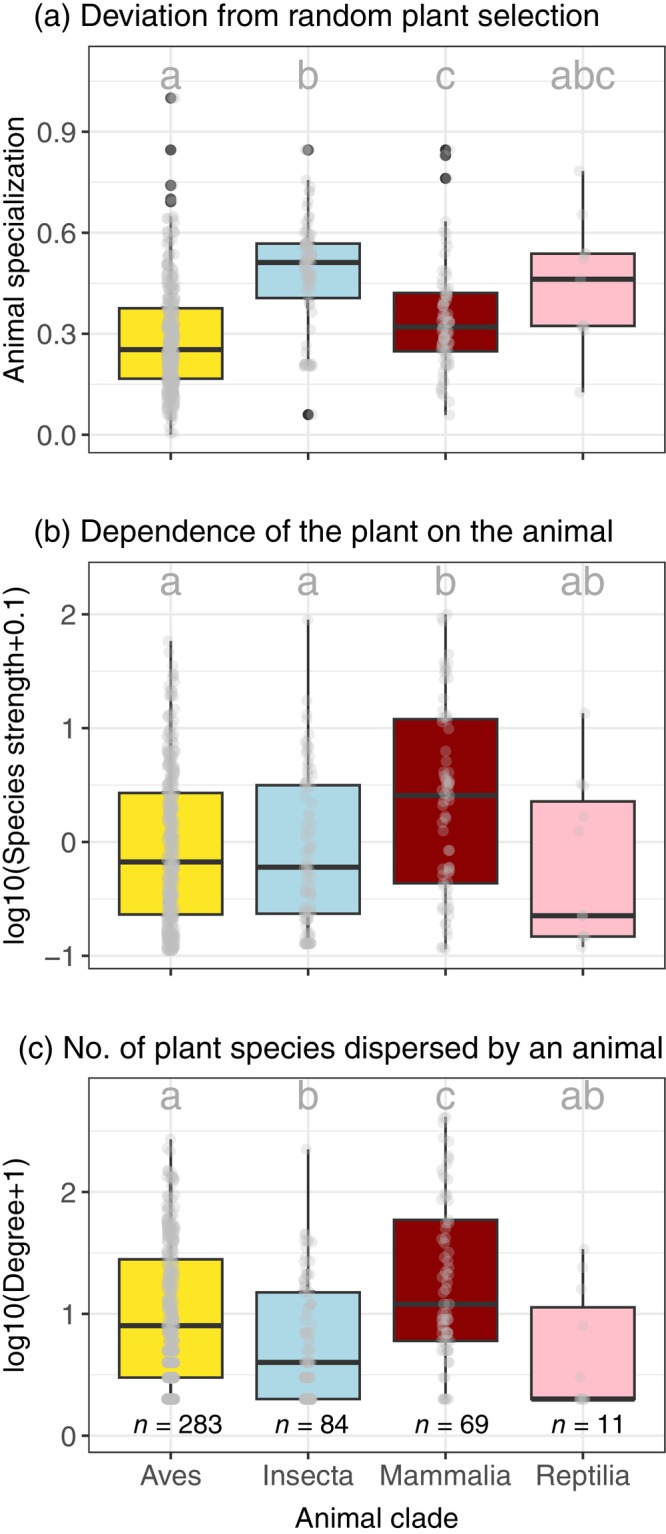
The roles of birds, insects, mammals, and reptiles in the European seed‐dispersal interaction network including all four biotic dispersal mechanisms. Boxplots depict median values with quantiles. Each gray dot is one animal species that interacts with multiple plant species via one of the four dispersal mechanisms, and animal clades that do not share the same lower letter (in gray) are significantly different based on generalized least squares analyses followed by multiple comparisons of means. Shown are (a) the level of animal specialization (d'*j*), which is how strongly an animal species deviates from a random sampling of the available interacting plant partners, ranging from 0 (no specialization) to 1 (perfect specialist); (b) the animal species strength, which is a measure of the importance of animals for plants; and (c) the animal degree, which reflects the number of plant species dispersed by each animal species.

Animals of the four clades differed in their topological roles within the network structure, as indicated by species‐level network metrics (Figure 2; Appendix S1: Table S2a). Insects were significantly more selective in their interactions with plants compared to birds and mammals, as they deviated more from random plant species selection than birds (higher animal specialization, GLS: *F*_3,442_ = 28.64, *p* < 0.001, Figure 2a). Mammal species were marginally (*p* = 0.046) more selective than birds (Figure 2a). Mammal species were on average more important for plant dispersal than birds and insects, as reflected by a higher animal species strength (GLS: *F*_3,442_ = 5.33, *p* = 0.001, Figure 2b). Similarly, mammal species dispersed on average more plant species than birds, insects and reptiles, and birds dispersed more plant species than reptiles (i.e., higher animal degree, GLS: *F*_3,442_ = 10.94, *p* < 0.001, Figure 2c).

The total contribution of each animal clade for the dispersal of all plant species, indicated by their cumulative species strength, was 863 for birds, 753 for mammals, 258 for insects, and 23 for reptiles. These values correspond to an estimated dependence of the entire flora on each of these groups of 45%, 40%, 14%, and 1.2%, respectively. Cumulative species strength was significantly higher for birds than for mammals (*X* ^*2*^(1) = 12.8, *p* < 0.001), for mammals than for insects (*X* ^*2*^(1) = 329.0, *p* < 0.001), and for insects than for reptiles (*X* ^*2*^(1) = 204.0, *p* < 0.001). Nearly half of the plant species were dispersed by multiple animal clades, namely 595 plants dispersed by two animal clades, 183 species dispersed by three clades, and 10 species dispersed by all four clades (Appendix S1: Table S3).

### Dry versus fleshy‐fruited plants

Most plant species that interacted with animals in Europe are dry‐fruited (85%, *X* ^*2*^(1) = 547.9, *p* < 0.001). The proportion of dry‐fruited species dispersed by each clade varied between 95% for insects (*n* = 455 species, including many with elaiosomes), 85% for mammals (*n* = 1156, lower than insects, *X* ^*2*^(1) = 25.6, *p* < 0.001), 82% for birds (*n* = 1208, *X* ^*2*^(1) = 3.8, *p* = 0.049), and only 56% for reptiles (*n* = 73, *X* ^*2*^(1) = 39.1, *p* < 0.001).

**FIGURE 3:**
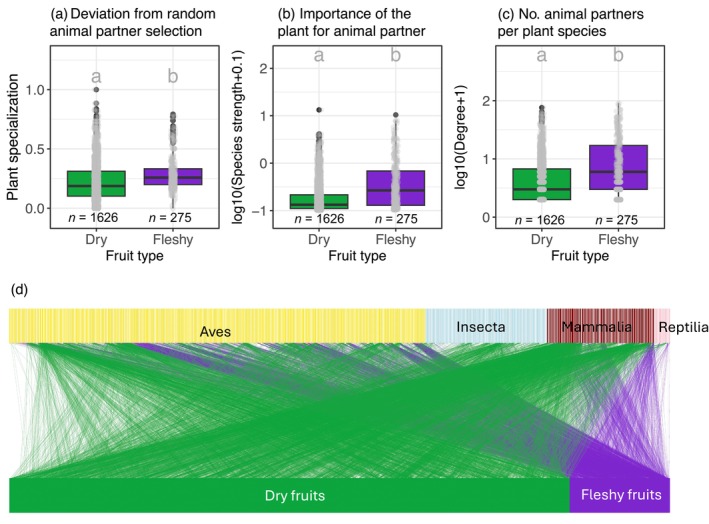
The roles of fleshy‐fruited and dry‐fruited plants in the seed‐dispersal interaction network including all animals and biotic interaction mechanisms. Shown are (a) the level of plant specialization d'_*i*_, which is how strongly the plant species deviates from a random sampling of the available interacting animal partners, ranging from 0 (no specialization) to 1 (perfect specialist); (b) the plant species strength, which is a measure of the importance of plants for the animal dispersers; and (c) the number of animal disperser species for each plant species (i.e., plant degree). Each gray dot is one plant species that interacts with multiple animal species via one of the four dispersal mechanisms, and fruit types that do not share the same lower letter (in gray) are significantly different based on generalized least squares analyses (panels a, b and c). (d) The species interaction network among European plant species and vector animals for interactions for plants with dry fruits (1626 species, green) and plants with fleshy fruits (275 species, purple) interacting with 283 bird, 84 insect, 69 mammal, and 11 reptile species. Each vertical bars represents one species.

Plants with fleshy fruits differed from those with dry fruits in their roles in the plant–animal interaction networks (Figure 3; Appendix S1: Table S2b); namely, fleshy‐fruited plants deviated more from random animal partner selection (higher plant specialization d'i metric, GLS: *F*_1,1899_ = 67.20, *p* < 0.001, Figure 3a), were on average more important for their animal partners (i.e., had a higher plant species strength, GLS: *F*_1,1899_ = 94.03, *p* < 0.001, Figure 3b), and had more animal partners per plant species (i.e., higher plant degree, GLS: *F*_1,1899_ = 67.20, *p* < 0.001, Figure 3c).

### Assigned syndromes versus observed dispersal mechanisms

**FIGURE 4:**
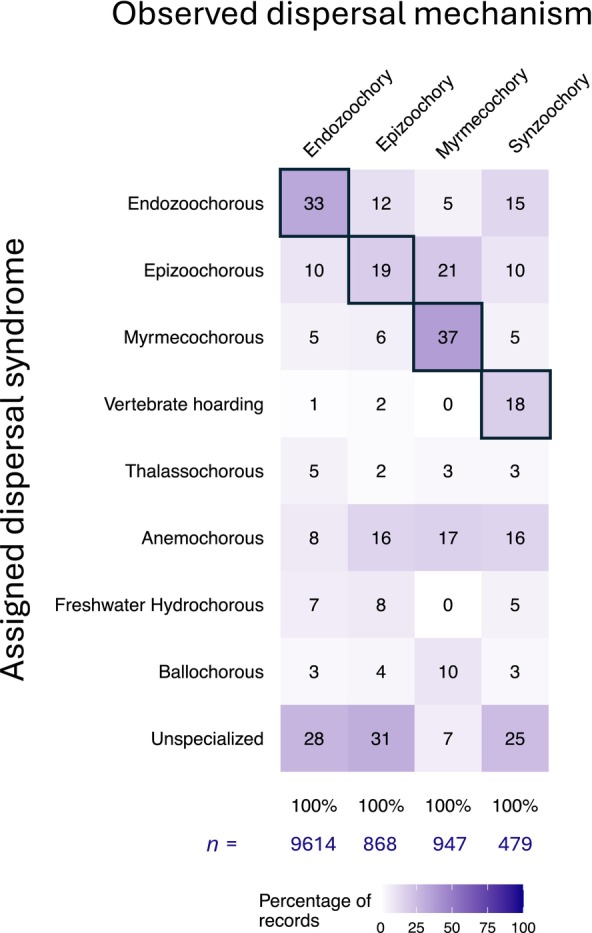
Comparison of the assigned dispersal syndrome based on species traits from the EuDis database (shown in rows) to the observed dispersal mechanisms in our database (shown in columns). The percentage assignment of each syndrome is calculated per observed mechanism, with the number of plant–animal interactions for each mechanism indicated below as *n*. Boxes with a black contour indicate “predicted dispersal” when assigned syndromes match observed mechanisms. Color intensity increases with a higher contribution of a syndrome to dispersal via the mechanism. Separate comparisons between syndromes and mechanisms for each animal clade can be found in Appendix S1: Figure S1.

Of the 1901 plant species in the database, 48% (*n* = 914) had one assigned syndrome, 11% (*n* = 201) had multiple assigned syndromes, and 41% (*n* = 786) had no specialized syndrome (i.e., unspecialized in terms of seed dispersal). Plant species were dispersed more frequently by the mechanism predicted based on their assigned dispersal syndrome than by a different mechanism (Omnibus chi‐square test of independence: expected = 25%, observed = 32%, *X* ^*2*^(1) = 334.9, *P* = < 0.001, Figure 4). Plants dispersed by a myrmecochory mechanism showed the highest matching rate with the assigned syndrome (37%), followed by endozoochory (33%), epizoochory (19%), and synzoochory (18%, Figure 4). However, in absolute terms, plant–animal interactions via mechanisms predicted by the syndromes are outnumbered by those dispersed by other (i.e., unpredicted) mechanisms. Importantly, many plant species with abiotic dispersal syndromes or with no identifiable syndromes according to EuDiS were also dispersed by animals, mainly by endozoochory, but also by the other three biotic mechanisms (Figure 4).

These patterns differed among the four animal clades (Appendix S1: Figure S1). Omnibus chi‐square tests of independence: Aves: expected = 37%, observed = 38%, *X* ^*2*^(1) = 6.0, *p* = 0.014; Mammalia: expected = 17%, observed = 21%, *X* ^*2*^(1) = 56.9, *p* < 0.001; Insecta: expected = 36%, observed = 37%, *X* ^*2*^(1) = 0.40, *p* = 0.53; Reptilia: expected = 11%, observed = 4%, *X* ^*2*^(1) = 53.5, *p* < 0.001. Notable variation among animal clades was that the epizoochory mechanism in mammals seems to be mostly explained by the presence of epizoochorous traits (20%), while for birds, it is mostly associated with the presence of endozoochorous traits (e.g. fleshy pericarp; 65%). Endozoochory was largely associated with endozoochorous traits for both insects (100%, but only based on two interactions, Appendix S1: Figure S1) and reptiles (52%). Mammals are relatively important vectors for ‘unspecialized’ seeds.

## DISCUSSION

Most seed dispersal studies to date have focused on a single dispersal mechanism, a single animal clade or on a small group of plants (Gómez et al., 2019; Green et al., 2023; Huang et al., 2025; Morán‐López et al., 2025; Quintero et al., 2024; van Leeuwen et al., 2023). In this study, we synthesized seed dispersal interactions across the four main clades of animal dispersers, which revealed that more than 350 bird and mammal species together disperse at least 17% (over 1700 plant species) of the total European flora (9874 species, Vargas et al., 2023). About half of these plant species are dispersed by both mammals *and* birds, while the other half relies on either mammals *or* birds. Overall, we estimated that 45% of the seed dispersal services are provided by birds, 40% by mammals, 14% by insects, and only 1% by reptiles. However, the contributions of birds to overall seed dispersal services in Europe stem mostly from the number of bird species dispersing seeds, while the contribution of mammals emerged directly from the high number of plants dispersed by each disperser species (on average more important than bird species). The integration of data across multiple animal clades, plant types, and dispersal mechanisms provides a new and broad perspective on seed dispersal services, which is essential to further advance our understanding of the role of animals for global seed dispersal dynamics (Fricke et al., 2022; Ozinga et al., 2009).

### Differential contribution of animal clades to dispersal mechanisms

Most animal‐dispersed European plant species are dispersed internally by animals (endozoochory), with considerably lower numbers of interactions through myrmecochory, epizoochory, or synzoochory. Epizoochory is almost exclusively carried out by mammals, and the role of birds, insects, and reptiles for epizoochory is relatively negligible, despite much speculation in historical literature about the importance of avian epizoochory (Green & Wilkinson, 2024). Synzoochory is performed chiefly by insects (i.e., myrmecochory), followed by birds (e.g., corvids) and, to a lesser extent, by mammals (e.g., rodents). Mammals and birds disperse most plant species via endozoochory, although their contributions are somewhat different. While mammals disperse more plant species than birds per animal species (higher animal degree), there are more than four times as many bird species dispersing seeds. This makes birds the most important seed dispersing clade in Europe, in terms of the cumulative dependence of the European animal‐dispersed flora on animal vectors.

The prominent role of birds and mammals relative to insects and reptiles might be related to a sampling bias and disproportionate focus of researchers on these easy‐to‐study and abundant groups. After controlling for the low sample size of reptiles in the analyses, significant differences with other clades disappeared. Despite these potential sampling biases, an important role of epizoochory in mammals may be explained by the overall higher *per species* importance of mammals for seed dispersal. Mammals have relatively large bodies with large surface areas suitable for seed adhesion (Costa et al., 2014; Sato et al., 2023). Like pollen and mud, seeds stick more easily to fur than to feathers, and birds spend much time preening and bathing to facilitate flight (Green & Wilkinson, 2024; Muchhala & Thomson, 2010). These observations are broadly in line with the much higher prevalence of endo‐ than epizoochory in comparative studies in birds (Brochet et al., 2010; Costa et al., 2014) and contrast with the historical underestimation of the capacity of dry‐fruited seeds to disperse by endozoochory (Green & Wilkinson, 2024). Interestingly, most of the relatively few plants whose seeds were dispersed by avian epizoochory had an endozoochory syndrome (Appendix S1: Figure S1).

In contrast to birds and mammals, which disperse seeds via multiple mechanisms, reptiles were only associated with dispersal via endozoochory, while insects disperse seeds almost exclusively via myrmecochory (the latter largely by definition, since this term is applied only to synzoochory by ants). Under this view, approximately two‐thirds of all synzoochory is performed by ants (i.e., myrmecochory), while one‐third is performed by birds and mammals. Our separation of individual dispersal mechanisms reveals the greater specialization metric of seed dispersal interactions by insects (and potentially reptiles). Insects and reptiles typically have fewer plant partners, and are often deemed less important for the dispersal of plant species, which is reflected in the number of studies including them. However, the role of insects and reptiles might also be particularly understudied compared to birds and mammals (Albert, Mårell, et al., 2015; Giladi, 2006; Green et al., 2023; Valido & Olesen, 2019; van Leeuwen et al., 2020, Mendes et al., 2026), and they might contribute via unique, presently unknown plant–animal interactions. Insects are often tightly linked as vectors to specific plant species, and can disperse species not dispersed by other clades (e.g., Pérez‐Cembranos et al., 2018; Appendix S1: Table S3).

### Dispersal of plants with dry and fleshy fruits

There is a strong and unfortunate segregation between studies on either dry or fleshy fruits dispersed by animals in the literature (van Leeuwen et al., 2022). By including plants with both dry and fleshy fruits in one large dataset, we found that dry‐fruited plant species represent 85% of the plants in the European zoochory interactions. This is not surprising since 92% of the European native plant species do not produce fleshy fruits (Vargas et al., 2023). However, even if fleshy‐fruited species are overrepresented in the European seed dispersal networks, ignoring dry‐fruited species means ignoring the large majority of the seed dispersal interactions. Fleshy‐fruited plants have more animal dispersers (higher plant degree) and are more important for animal dispersers (higher plant species strength). This suggests that plant species with fleshy fruits provide more attractive, or higher quality, resources for animal dispersers (see e.g., Short & Epps, 1976 for a study comparing the nutrient content in dry and fleshy fruits), yet there are (quantitatively) more interactions between animal species that disperse dry‐fruited plant species by endozoochory across all clades and mechanisms. Plants with fleshy fruits are dispersed by specific subsets of the whole disperser's assemblage, while dry‐fruited plants tend to be dispersed by a less selective proportion of the dispersal vectors identified in this study.

Taken altogether, these results suggest that fleshy fruits are more attractive to frugivorous animals and therefore more actively selected than dry fruits—which are perhaps consumed more proportionally to their abundance (Huang et al., 2025; Peña et al., 2023). However, alternatively, dispersal of dry fruits may also be related to traits that are currently not included in dispersal syndromes, and hence not researched and interpreted similarly. More research on these ideas is needed, as only 8% of the native European flora actually produces fleshy fruits (Vargas et al., 2023), and these have received most research attention. In our study, the number of vectors for dry‐fruited plants may therefore have been underestimated (Beckman & Sullivan, 2023). Still, the broad‐scale observed variation between dry and fleshy plants may have important implications in the light of vulnerability of the interactions to global change and defaunation (Dirzo et al., 2014; Ozinga et al., 2009). If plants with fleshy fruits generally have more animal partners, this may reduce their risk of losing a large number of their animal dispersers simultaneously. Plants with dry fruits may additionally be dispersed more by alternative abiotic dispersal mechanisms, although those mechanisms likely provide shorter dispersal distances than those provided by birds and mammals (Bullock et al., 2017; Wasowicz et al., 2025).

### Assigned syndromes versus observed dispersal mechanisms

We evaluated the predictive power of dispersal syndromes to infer observed dispersal interactions. As expected, syndromes increased the probability of dispersal by a specific mechanism (see the diagonal on Figure 4). Indeed, the biotic syndromes matched the observed mechanisms more than what can be expected randomly, as a random expectation might have been to have 11% of the plant–animal interactions for each of the possible syndromes for every mechanism, adding up to 100% of the interactions. However, if syndromes would consistently predict the mechanisms, the diagonal of the biotic syndromes would have been 100%. In reality, the syndromes correctly predicted observed dispersal for between 18 and 37% of the cases, which is more than expected at random, but well below 100%.

In absolute terms, more dispersal interactions were recorded between animals and plant species without biotic dispersal syndromes than between animals and plant species with biotic dispersal syndromes. This is indicated by the values in the bottom five rows in Figure 4. Endozoochory, epizoochory, and synzoochory notably contributed to the dispersal of ‘unspecialized’ plant species, which had a similar or better match with these mechanisms than seeds with their matching syndromes (Figure 4). Animals dispersed many seeds of plant species with anemochorous syndromes, which suggests that certain diaspore traits (e.g., pappi) may have a positive effect on biotic dispersal (Costa et al., 2014). Overall, the large proportion of species dispersed by animals without traits currently considered diagnostic for promoting biotic dispersal shows that inferring dispersal mechanisms based on traits alone provides a very incomplete view of the seed dispersal process (Almeida et al., 2025; Correia et al., 2018; Green et al., 2022; Vargas et al., 2012). Accordingly, inferring vectors directly from syndromes is problematic if one assumes a purely deterministic action of evolution (Costa et al., 2014; Green et al., 2022; Heleno et al., 2011), and such an approach does not acknowledge that there is inter‐ and intraspecific variation that drives the functioning of living systems.

Certain syndromes were found to be more linked to certain clades than to others (Appendix S1: Figure S1). Birds and reptiles, for example, disperse a high proportion of seeds with an endozoochorous syndrome via endozoochory, while the epizoochorous syndrome is better able to predict dispersal by epizoochory for mammals than for the other three clades. The vertebrate hoarding syndrome predicts dispersal via synzoochory for mammals better than it does for birds, although for both clades synzoochory is more recorded for ‘unspecialized’ seeds. Especially for plants dispersed by mammals, many species are considered ‘unspecialized’, yet they may possess understudied traits (e.g., hard, small, round seeds, D'hondt, 2011) that could have evolved for other purposes (e.g., seed bank persistence, fungal avoidance, Bruun & Poschlod, 2006, Albert, Auffret, et al., 2015).

### Conclusions and implications

Analyzing a Europe‐wide, multi‐clade seed dispersal database identified birds and mammals as the most dominant seed dispersing clades in Europe, with a greater richness of bird dispersers but a greater contribution to seed dispersal per mammal species. Animal clades are linked to specific dispersal mechanisms, with epizoochory almost exclusively carried out by mammals, synzoochory mostly carried out by insects (myrmecochory), and endozoochory by birds and mammals. Most seed dispersal interactions by all clades and mechanisms involve dry fruits, while fleshy‐fruited plants tend to be dispersed by more animal species. While syndromes offer some moderate predictive power over the more likely dispersal mechanisms, inferring seed dispersal mechanisms based on diaspore traits alone is unjustified. Syndromes only predict the actual mechanisms in roughly one‐third of the pairwise interactions, and animals disperse many plant species assigned abiotic or unspecialized syndromes. These Europe‐wide patterns call for careful use of dispersal syndromes in global studies of plant–animal interactions.

## AUTHOR CONTRIBUTIONS

Conceptualization (all authors as CONNECT consortium), data collection (Sara B. Mendes, Ruben Heleno), data analysis (Casper H. A. van Leeuwen, Evan Fricke, Sara B. Mendes), writing first draft (Casper H. A. van Leeuwen), and editing and revising manuscript (all authors).

## CONFLICT OF INTEREST STATEMENT

All authors declare no conflicts of interest.

## Supporting information


Appendix S1:

## Data Availability

Data (Mendes & van Leeuwen, 2026) are available in Figshare at https://doi.org/10.6084/m9.figshare.31268224.v4.
