# Supplementary material for: Fuzzy relationships among plant dispersal mechanisms, syndromes, and animal vectors
Source: Ecology. 2026 Jul 8;107(7):e70450. doi: 10.1002/ecy.70450 (PMC13343317; doi:10.1002/ecy.70450)
Supplement: Supplementary file 1 — Appendix S1: [file ECY-107-e70450-s001.pdf]

Appendix S1

Fuzzy relationships among plant dispersal mechanisms, syndromes, and animal vectors

Casper H.A. van Leeuwen, Ruben Heleno, Andy J. Green, Esther Sebastián-González, José Miguel Costa, Christophe Baltzinger, Evan Fricke, Ádám Lovas-Kiss, Irene Castañeda, Isabel Donoso, Alistair G. Auffret & Sara B. Mendes

Ecology

Table S1: Sample coverage of plants, animals and their interactions for each animal clade. Sample coverage was estimated using the R-package *iNext* (Hsieh et al, 2024). We built incidence matrices for each clade (birds, insects, mammals and reptiles). In each matrix, columns were individual bibliographic references and rows were animal species, plant species, and unique pairwise interactions, respectively.

**Type of**	**Clade**	**Sample coverage**
Plants	Birds	94%
Plants	Insects	52%
Plants	Mammals	92%
Plants	Reptiles	70%
Animals	Birds	97%
Animals	Insects	90%
Animals	Mammals	97%
Animals	Reptiles	94%
Interaction	Birds	70%
Interaction	Insects	28%
Interaction	Mammals	66%
Interaction	Reptiles	60%

Table S2: Results of General Least Squares modelling with implemented variance identity structures (varIdent) by groups. Models test for (**a**) difference among animal clades, and (**b**) differences between plants with fleshy or dry fruits. The first mentioned factor levels are set as intercepts.

	Model	dependent variable	factor	Contrast		estimate	SE	df	t ratio	P value
**(a)**	M1	Animal specialization d'j	Clade	Aves	Insecta	-0.192	0.021	140.10	-9.03	<0.001
				Aves	Mammalia	-0.060	0.022	105.30	-2.71	0.046
				Aves	Reptilia	-0.158	0.057	10.30	-2.77	0.12
				Insecta	Mammalia	0.133	0.027	143.50	4.91	<0.001
				Insecta	Reptilia	0.034	0.059	11.90	0.58	1.00
				Mammalia	Reptilia	-0.098	0.059	12.20	-1.66	0.74
	M2	Animal species strength	Clade	Aves	Insecta	0.025	0.083	133.70	0.30	1.00
				Aves	Mammalia	-0.416	0.108	90.50	-3.86	<0.001
				Aves	Reptilia	0.150	0.220	10.10	0.68	1.00
				Insecta	Mammalia	-0.441	0.124	128.70	-3.57	<0.001
				Insecta	Reptilia	0.125	0.228	11.70	0.55	1.00
				Mammalia	Reptilia	0.565	0.238	13.90	2.38	0.20
	M3	Animal degree	Clade	Aves	Insecta	0.240	0.061	154.40	3.90	<0.001
				Aves	Mammalia	-0.254	0.089	88.60	-2.84	0.03
				Aves	Reptilia	0.345	0.152	10.80	2.26	0.27
				Insecta	Mammalia	-0.494	0.098	115.30	-5.03	<0.001
				Insecta	Reptilia	0.105	0.158	12.40	0.67	1.00
				Mammalia	Reptilia	0.599	0.171	16.70	3.51	0.02
**(b)**	M4	Plant specialization d'i	Dry or fleshy	Dry	Fleshy	-0.242	0.030	322.00	-8.20	<0.001
	M5	Plant species strength	Dry or fleshy	Dry	Fleshy	-0.297	0.031	309.00	-9.70	<0.001
	M6	Plant degree	Dry or fleshy	Dry	Fleshy	-0.242	0.030	322.00	-8.20	<0.001

Table S3: The number of plant species uniquely dispersed by each combinations of the four different clades. The number in the final column denotes the number of species that is dispersed by all clades mentioned in the left columns. Note that the number of plant species in the matrix also increases if more animal species are included.

Clade				Number of species dispersed exclusively by this/these clade(s)
Reptilia				11
Mammalia				432
Mammalia	Reptilia			6
Insecta				165
Insecta	Reptilia			7
Insecta	Mammalia			66
Insecta	Mammalia	Reptilia		6
Aves				505
Aves	Reptilia			8
Aves	Mammalia			462
Aves	Mammalia	Reptilia		22
Aves	Insecta			46
Aves	Insecta	Reptilia		3
Aves	Insecta	Mammalia		152
Aves	Insecta	Mammalia	Reptilia	10

Observed dispersal mechanism (columns); **Assigned dispersal syndrome** (rows)

	All clades				*Aves*				*Mammalia*				*Insecta*				*Reptilia*			
	Endo	Epi	Myr	Syn	Endo	Epi	Myr	Syn	Endo	Epi	Myr	Syn	Endo	Epi	Myr	Syn	Endo	Epi	Myr	Syn
Endozoochorous	33	12	5	15	40	65	0	17	22	8	0	10	100	0	5	8	52	0	0	0
Epizoochorous	10	19	21	10	9	6	0	11	11	20	0	9	0	0	21	8	7	0	0	0
Myrmecochorous	5	6	37	5	5	2	0	6	5	6	0	4	0	0	37	15	5	0	0	0
Vertebrate hoarding	1	2	0	18	1	0	0	14	2	2	0	26	0	0	0	31	0	0	0	0
Thalassochorous	5	2	3	3	6	5	0	4	3	2	0	0	0	0	3	0	6	0	0	0
Anemochorous	8	16	17	16	7	10	0	17	11	17	0	12	0	0	17	15	10	0	0	0
Freshwater Hydrochorous	7	8	0	5	8	15	0	6	5	8	0	1	0	0	0	0	1	0	0	0
Ballochorous	3	4	10	3	3	0	0	3	4	4	0	2	0	0	10	0	7	0	0	0
Unspecialized	28	31	7	25	21	0	0	22	37	33	0	36	0	0	7	23	12	0	0	0
	100%	100%	100%	100%	100%	102%	100%	100%	100%	100%	100%	100%	100%	100%	100%	106%	100%	100%	100%	100%
n =	9614	868	947	479	6065	62	0	326	3461	806	0	140	2	0	947	13	86	0	0	0

Figure S1: Comparing the assigned dispersal syndrome based on species traits from the EuDis database (shown in rows) to the observed dispersal mechanisms in our database (shown in columns) with number of plant-animal interactions indicated below as n. The percentage assignment of each syndrome is calculated per mechanism, and color intensity increases with a higher contribution of that syndrome. Comparisons between syndromes and mechanisms are indicated for all four animal clades, and separately colored per animal clade. Omnibus chi-square tests of independence: Aves: expected = 37%, observed = 38%, $X^2(1) = 6.0$, P = 0.014; Mammalia: expected = 17%, observed = 21%, $X^2(1) = 56.9$, P < 0.001; Insecta = expected = 36%, observed = 37%, $X^2(1) = 0.40$, P = 0.53; Reptilia = expected = 11%, observed = 4%, $X^2(1) = 53.5$, P < 0.001). Note that because some plant species have multiple assigned dispersal syndromes, total percentages can exceed 100% (i.e., one plant-animal interaction can contribute multiple links between one mechanism and multiple syndromes).

References

Hsieh T, Ma K, Chao A. 2024. iNEXT: Interpolation and Extrapolation for Species Diversity. Version 3.0.1.
